# Immunomodulatory and Prebiotic Effects of 2′-Fucosyllactose in Suckling Rats

**DOI:** 10.3389/fimmu.2019.01773

**Published:** 2019-07-31

**Authors:** Ignasi Azagra-Boronat, Malén Massot-Cladera, Jordi Mayneris-Perxachs, Karen Knipping, Belinda van't Land, Sebastian Tims, Bernd Stahl, Johan Garssen, Àngels Franch, Margarida Castell, M. José Rodríguez-Lagunas, Francisco J. Pérez-Cano

**Affiliations:** ^1^Physiology Section, Department of Biochemistry and Physiology, Faculty of Pharmacy and Food Science, University of Barcelona, Barcelona, Spain; ^2^Nutrition and Food Safety Research Institute (INSA-UB), Santa Coloma de Gramenet, Spain; ^3^Eurecat, Centre Tecnològic de Catalunya, Technological Unit of Nutrition and Health and Technological Unit of Omic Sciences, Reus, Spain; ^4^Danone Nutricia Research, Utrecht, Netherlands; ^5^Division of Pharmacology, Faculty of Science, Utrecht Institute for Pharmaceutical Sciences, Utrecht University, Utrecht, Netherlands; ^6^Laboratory of Translational Immunology, University Medical Centre Utrecht, Wilhelmina Children's Hospital, Utrecht, Netherlands

**Keywords:** 2′-FL, microbiota, HMO, immunoglobulins, metabolomics

## Abstract

Human milk oligosaccharides are unconjugated complex glycans present in high concentration in human milk that serve as pre-biotics and immunomodulators. They are not primarily absorbed or metabolized by the infant and reach the lower part of the intestinal tract unaltered. One of the main oligosaccharides found in human milk is 2′-fucosyllactose (2′-FL). This study aimed to investigate the effects of daily oral administration of 2′-FL in healthy suckling rats. From days 2 to 16 of life, rats were daily given the oligosaccharide (2′-FL) or vehicle (REF), weighed and their stool characteristics were assessed. On days 8 and 16 of life the morphometry, intestinal architecture, and cytokine release, mesenteric lymph nodes cell composition, plasma immunoglobulin concentrations, fecal microbiota composition, cecal short-chain fatty acids content, and the urinary metabolic profile were assessed. Animals given 2′-FL showed higher plasma IgG and IgA and more T cell subsets in the mesenteric lymph nodes on day 16. Moreover, at intestinal level, villus heights, and areas were increased on day 8. Cecal samples displayed a higher *Lactobacillus* proportion and a different urinary metabolic profile was observed on day 8, and a higher proportion of butyrate on day 16. In conclusion, supplementation of 2′-FL in early life has a pre-biotic and intestinal trophic effect and promotes maturation of the immune system.

## Introduction

Human milk provides all the essential nutrients to newborn and developing infants, as well as bioactive compounds such as human milk oligosaccharides (HMOs), which promote health benefits beyond nutrition (1). HMOs are produced in the mammary gland from the monosaccharides galactose, glucose, N-acetylglucosamine, fucose, and sialic acid, forming unconjugated complex glycans consisting of both short chain as well as long chain structures (in 9:1 ratio), that range from 10 to 15 g/L in mature milk (2). The presence of fucose and sialic acid renders the HMOs resistant to digestion, thus allowing them to reach the large intestine of an infant unaltered (2, 3). Moreover, ~1% of the ingested HMOs are absorbed, reach the systemic circulation and are excreted in urine without being metabolized (2).

To date, more than 200 HMOs having 3–22 monosaccharide units have been identified. The secretor and the Lewis blood group status direct the amount and type of oligosaccharides present in the milk (4). In this respect, 2′-fucosyllactose (2′-FL) is the major short chain HMO found in the milk of secretor (FUT2)-positive women, accounting for more than 30% of the total HMOs mixture (5). The concentration of 2′-FL in human milk remains stable in the first month post-delivery; afterwards, its concentration declines (3).

2′-FL has been demonstrated to confer beneficial effects both *in vitro* and *in vivo*. These effects can be classified into two groups, depending on whether they interact directly with host cells and tissues or indirectly through their action on the gut microbiota or pathogens (3). It has been shown that 2′-FL has anti-inflammatory potential, for example by modulating CD14 expression in human enterocytes and thus quenching inflammatory signaling (5), or by reducing the concentration of plasma inflammatory cytokines in infants (6). Numerous intestinal pathogens employ the host oligosaccharide sequences found on the cell surface of enterocytes to adhere as the first step in the infection process. Because HMOs, such as 2′-FL, also have a carbohydrate structure, they can act as soluble receptor analogs and prevent the pathogen from infecting the host (7). In this regard, 2′-FL has been shown to inhibit the adhesion or infectivity of microbes, namely *Campylobacter jejuni* (8), Enteropathogenic *Escherichia coli* (9), rotavirus (10), and norovirus (11). In addition, supplementation of infants with HMOs promotes a healthy microbiome dominated by bifidobacteria and *Bacteroidetes*, which have the specific glycosidases to utilize them as a source of energy. Contrarily, most pathogenic *Enterobacteriaceae* do not have these enzymes and thus, their growth is inhibited (12). Specifically, 2′-FL has been shown to be a preferred substrate for bacteria isolated from human gut, such as *Bifidobacterium longum* subsp*. infantis, Bifidobacterium bifidum, Bacteroides fragilis*, and *Bacteroides vulgatus* (13, 14). Accordingly, these bacteria produce short-chain fatty acids (SCFAs) and other metabolites that may lower the gut pH, impacting the composition of microbial communities and limiting the growth and colonization of harmful microorganisms, such as some pathogenic species of bacteroides, clostridia and coliforms (3, 15–17).

A great part of the literature focuses on assessing the effects of complex HMO mixtures, thus not enabling their individual properties to be deciphered. In fact, there are only a few clinical studies that have assessed the effects of the supplementation of formulas with 2′-FL (18, 19). These studies provide evidence that supplementation with 2′-FL is safe, well-tolerated and that the infant's growth is comparable with that of a breastfed infant. However, there is limited evidence on the specific health improvements of 2′-FL and its mechanism of action. Moreover, although 2′-FL seems to be a good candidate to be considered as a pre-biotic, there is still not enough data to support this (20).

Therefore, the present study aimed to investigate the effects of daily supplementation with 2′-FL in healthy suckling rats. To achieve this, we assessed intestinal and systemic parameters in relation to animal growth, immune system, intestinal architecture, microbiota composition, and metabolism.

## Materials and Methods

### Animals

Pregnant Lewis rats (LEW/OrlRj, *N* = 6) with 15 days of gestational age (G15) were obtained from Janvier Labs (Le Genest-Saint-Isle, France) and individually housed in cages (2184L Eurostandard Type II L, Tecniplast, West Chester, PA, USA) containing bedding of large fibrous particles (Souralit 1035, Bobadeb S.L., Santo Domingo de la Calzada, Spain) and tissue papers (Pañuelos desmaquillantes, Gomà-Camps S.A.U., La Riba, Spain). Pregnant rats were monitored daily and allowed to deliver at term. The day of birth was established as day 1 of life. On day 2, litters were randomly assigned to the experimental groups (three dams with their litters/group) and culled to eight pups per lactating dam, with a similar number of each sex in each litter, and with free access to maternal milk and rat diet. Dams were given a standard diet corresponding to the American Institute of Nutrition 93G formulation (21) (Teklad Global Diet 2014, Envigo, Indianapolis, IN, USA) and water *ad libitum*. To avoid influence of biological rhythms, animal handling was performed on a scheduled basis during the first hours of the light phase. Daily handling and oral administration were performed after separating all the mothers and keeping the pups in the home-cage. Afterwards, the dam was reunited with the whole litter. Animals were housed under controlled conditions of temperature and humidity in a 12 h light−12 h dark cycle, in the Faculty of Pharmacy and Food Science animal facility (University of Barcelona, Spain). All experimental procedures were conducted in accordance with the institutional guidelines for the care and use of laboratory animals and were approved by the Ethical Committee for Animal Experimentation of the University of Barcelona and the Catalonia Government (CEEA-UB Ref. 74/05 and DAAM 3046, respectively), in full compliance with national legislation following the EU-Directive 2010/63/EU for the protection of animals used for scientific purposes.

### Experimental Design and Sample Collection

Neonatal rats and their respective dam were randomly distributed into two groups (3 litters in each group). In the 2′-FL group, pups received by oral gavage 0.2 g of 2′-FL/100 g of body weight (4.5 μL/g/day) from day 2; and in the REF group pups received the same volume of mineral water (vehicle) also by oral gavage. 2′-FL was produced by microbial fermentation, with >90% purity and residual components in dry powder including glucose, fucose, lactose, 3′-FL, difucosyllactose, and water (provided by Danone Nutricia Research, The Netherlands). The dose of 2′-FL was selected in basis of the daily consumption per body weight of this particular HMO by a baby (22, 23) and previous studies (24, 25), which in addition has been demonstrated to be a safe dose in rats (26). Rat body weight was monitored daily. Moreover, the naso–anal and tail lengths were measured to determine the body/tail ratio. The body mass index (BMI) was calculated as body *weight/length*^2^ (g/cm^2^) and the Lee Index was calculated as (*weight*^0.33^*/length)* × *1,000* (g^0.33^/cm). Fecal samples were obtained daily after gentle abdominal massage to determine changes in fecal weight and consistency due to the supplementation with 2′-FL. Stool consistency was scored in a blinded manner based on texture and amount as follows: normal feces (1); soft feces (2); totally loose feces (3); high amount of watery feces (4). On days 8 and 16 of life, half of each litter (four randomly selected pups/dam) was euthanized to obtain tissue samples. The weight of spleen, thymus, liver, small intestine, and large intestine were recorded. Moreover, the length of the small and large intestines was measured. Mesenteric lymph nodes (MLNs) were obtained to study the proportion of specific immune cell populations. Plasma samples were used to determine the total systemic immunoglobulins (Igs). The small intestine was rinsed with PBS. The central part was obtained to quantify the relative gene expression of several genes and a fragment of the distal jejunum to study histomorphometric changes. The remaining parts of the small intestine were opened lengthwise, cut into 5 mm pieces, incubated with 2 mL of PBS in a shaker (10 min, 37°C) and then centrifuged in order to obtain the gut wash (GW), which enabled to quantify the intestinal cytokine release. Fecal samples were obtained on day 8 by abdominal massage for the analysis of the microbiota composition. Moreover, short-chain fatty acids (SCFAs) were analyzed in the cecal content, at the end of the study. Finally, for the metabolomic study, urine samples were post-mortem directly obtained from the bladder by puncture.

### Quantification of Immunoglobulins and Cytokines

The quantification of Ig in plasma (IgM, IgG1, IgG2a, IgG2b, IgG2c, and IgA) and cytokines in the gut wash (IL-1α, IL-4, IL-6, IL-10, IL-12p70, IFN-γ, and TNF-α) was performed on samples obtained in rats euthanized on days 8 and 16 of life, as previously described (24). Briefly, specific color-coded capture beads were bound to the analyte of interest. After adding different detection antibodies conjugated to phycoerythrin (PE), for the quantification of Ig, or biotin and adding afterwards streptavidin-PE for the quantification of cytokines, the specific concentration of each analyte was obtained by MAGPIX® analyzer (Luminex Corporation, Austin, TX, USA) at the Cytometry Service of the Scientific and Technological Centers of the University of Barcelona (CCiT-UB).

### Isolation of Mesenteric Lymph Node Cells

MLN cells of 16-day-old rats were obtained by passing the tissue through a sterile 40 μm mesh cell strainer (Thermo Fisher Scientific, Barcelona, Spain) placed on a Petri dish on an ice surface and containing Roswell Park Memorial Institute (RPMI) 1640 medium (Sigma-Aldrich, Madrid, Spain) enriched with 10% fetal bovine serum (FBS, Sigma-Aldrich), 100 IU/mL streptomycin-penicillin (Sigma-Aldrich), 2 mM L-glutamine (Sigma-Aldrich), and 0.05 mM 2-β-mercaptoethanol (Merck Millipore, Darmstadt, Germany). The suspension was centrifuged (538 *g*, 10 min, 4°C) and resuspended in the enriched RPMI. The number and viability of cells were determined by Countess™ Automated Cell Counter (Invitrogen™, Thermo Fisher Scientific).

### Immunofluorescence Staining and Flow Cytometry Analysis

MLN cell suspensions (3 × 10^5^ cells) were stained using fluorochrome-conjugated monoclonal antibodies (mAbs). The mouse anti-rat mAb conjugated to fluorescein isothiocyanate (FITC), phycoerythrin (PE), peridinin chlorophyll protein (PerCP), or allophycocyanin (APC) used here included anti-CD4 (OX-35), anti-CD8α (OX-8), anti-TCRαβ (R73), anti-TCRγδ (V65), and anti-NKR-P1A (10/78), all from BD Biosciences (San Diego, CA, USA); anti-CD45RA (OX-33) from Caltag (Burlingame, CA, USA); anti-CD8β (3·41) from Serotec (Kidlington, Oxford, UK); anti-αE integrin (OX-62) and anti-CD62L (OX-85) from BioLegend (San Diego, CA, USA). Staining was performed as previously described (27). Results were acquired using a Gallios™ flow cytometer (Beckman Coulter Inc., Madrid, Spain) in the Cytometry Service of the CCiT-UB. The data obtained were analyzed with FlowJo 10.0.7 software (Tree Star Inc., Ashland, OR, USA).

### Quantification of Intestinal Gene Expression

A portion of about 0.5 cm of the central section of the small intestine of 8-day-old rats was homogenized for 30 s in lysing matrix tubes (MP Biomedicals, Illkirch, France) using a FastPrep-24 instrument (MP Biomedicals), as previously described (28). RNA was isolated with the RNeasy® Mini Kit (Qiagen, Madrid, Spain) following the manufacturer's instructions. RNA purity and concentration were determined with a NanoPhotometer (BioNova Scientific S.L., Fremont, CA, USA). Later, cDNA was obtained using thermal cycler PTC-100 Programmable Thermal Controller and TaqMan® Reverse Transcription Reagents (Applied Biosystems, AB, Weiterstadt, Germany).

The specific PCR TaqMan® primers (AB) used to assess gene expression with real-time PCR (ABI Prism 7900 HT, AB) were *pIgR* (Rn00562362_m1, I), *Iga* (331943, made to order), *Tlr2* (Rn02133647_s1, inventoried [I]), *Tlr3* (Rn01488472_g1, I), *Tlr4* (Rn00569848_m1, I), *Tlr5* (Rn04219239_s1, I), *Tlr7* (Rn01771083_s1, I), *Tlr9* (Rn01640054_m1, I), *Ifng* (Rn00594078_m1, I), *Tnf* (Rn99999017_m1, I), *Tgfb1* (Rn00572010_m1, I), *Il10* (Rn00563409_m1, I), *Il17a* (Rn01757168_m1, I), *Il22* (Rn01760432_m1, I), *Muc2* (Rn01498206_m1, I), O*cln* (Rn00580064_m1, I), *Cldn2* (Rn02063575_s1, I), *Fcgrt* (Rn00583712_m1, I, encoding for FcRn), and *Prdm1* (Rn03416161_m1, I, encoding for Blimp-1). The relative gene expression was normalized to the housekeeping gene *Gusb* (Rn00566655_m1, I) using the 2^−ΔΔ*Ct*^ method (29). Results were expressed as the percentage of expression in each experimental group normalized to the mean value obtained for the REF group, which was set at 100%.

### Histomorphometric Analysis

Intestinal distal jejunum samples of rats sacrificed 8 days after birth were dehydrated, paraffin-embedded, sectioned, and stained with hematoxylin-eosin, as previously described (24). Intestinal architecture was analyzed by bright-field microscopy (Olympus BX41, Olympus Corporation, Shinjuku, Tokyo, Japan) at 100× magnification. The intestinal perimeter was calculated by measuring the outer layer of the intestine. Seven villi from each animal were randomly selected and villus heights and areas were measured. Villus widths were measured at the crypt-villus junction. Crypt depths and the ratio villus heights to crypt depths were calculated. Statistical analysis was performed with each animal's mean value of the parameters described above. All morphometric measurements were performed with ImageJ (Image Processing and Analysis in Java, National Institute of Mental Health, Bethesda, MD, USA).

### Fecal Microbiota Composition

Genomic DNA was extracted from day-8 fecal samples ranging 5–20 mg, using Qiamp DNA Stool Mini kit (Qiagen), enzymatic lysis and mechanic disruption (30). Extra purification and concentration were performed following the cleaning protocol from Qiamp Micro kit (Qiagen). Fifty ng of DNA were amplified following the 16S Metagenomic Sequencing Library Illumina 15044223 B protocol (Illumina Inc, San Diego, CA, USA) and sequences were merged and processed using Pair-End read merger (PEAR v 0.9.6, Exelixis Lab, Heidelberg, Germany) and Cutadapt v1.8.1 (31).

To estimate the specific biodiversity, the Shannon–Wiener and CHAO1 indexes were calculated. In order to study the presence or absence of taxonomic ranks (family, genera and species) in the experimental groups, it was agreed that all groups displaying two or three animals (out of the three randomly analyzed in each group) with some bacterial proportion were computed as present, while the groups displaying one animal or none were computed as absent. Venn diagrams and heat maps based on log_2_ fold change with respect to 2′-FL were represented. Moreover, principal components analysis (PCA) was conducted to search for natural clustering of the samples with Matlab (MathWorks, Natick, MA, USA) using in-house scripts.

### Quantification of Short-Chain Fatty Acids in the Cecal Content

The quantification of cecal SCFAs in neonatal rats of 16 days of life was performed by headspace-gas chromatography-mass spectrometry (HS-GC-MS) at the GC-MS unit of the CCiT-UB, as previously described (24). Acetic, propionic, isobutyric, butyric, isovaleric, valeric, isocaproic, caproic, and heptanoic acids were quantified.

### Metabolomic Analysis of Urine

Urine samples of 16-day-old rats (six random samples from each group obtained immediately after sacrifice) were thawed at room temperature and homogenized using a vortex mixer. Five hundred microliters of phosphate buffer (pH 7.4; 100% D_2_O) containing 1 mM of the internal standard, 3-trimethylsilyl-1-[2,2,3,3-2H4] propionate (TSP) were added to 100 μl of urine. Samples were mixed and centrifuged (10,000 *g*, 10 min, room temperature) before transfer to a 5.0 mm nuclear magnetic resonance (NMR) tube. All samples were analyzed on a 600 MHz Bruker Avance III NMR spectrometer equipped with a 5 mm PABBO BB-1H/D Z-GRD probe and a SampleJet robot (Bruker Biospin, Germany). Standard one-dimensional ^1^H NMR spectra of the urine samples were acquired using a standard one-dimensional NOESY pre-saturation pulse sequence (Bruker sequence code noesypr1d). Water resonances were suppressed with irradiation at the water frequency during both the recycle delay of 5 s and the mixing time of 100 ms. For each sample, a total of 256 scans were collected in 64K data points with a spectral width of 16 ppm and an acquisition time of 3.42 s. Two-dimensional ^1^H–^1^H correlation spectroscopy (COSY), ^1^H–^1^H total correlation spectroscopy (TOCSY), ^1^H–^13^C heteronuclear single quantum coherence (HSQC) and J-resolved NMR spectra were acquired to aid metabolite identification in addition to statistical total correlation spectroscopy (STOCSY). The free induction decay was multiplied by an exponential weighting function corresponding to 0.3 Hz line broadening prior to Fourier transform. Spectra were manually phased and corrected for baseline distortions using Topspin 3.5. All spectra were referenced to the TSP singlet at δ 0.0. ^1^H NMR spectra (δ 0.2–10.0) were digitized into consecutive integrated spectral regions (~20,000) of equal width (0.00055 ppm). The regions between δ 4.50–5.00 and 5.58–5.95, containing the residual water and urea peaks, respectively, were removed in order to minimize baseline effects caused by imperfect water suppression. Each spectrum was then normalized to unit area to account for variation in the sample concentration. All ^1^H NMR spectra were then aligned using a recursive segment-wise peak alignment method. Three urine samples contained a high amount of bladder tissue and they had to be discarded from the analysis.

PCA with mean centering and pareto scaling was performed in in Matlab using in-house scripts in order to visualize patterns, clusters and outliers within the data set (32). Then, ^1^H NMR spectroscopic profiles were used as the descriptor matrix (X) and class membership (e.g., REF or 2′-FL) was used as the response variable (Y) to perform an orthogonal projection to latent structures-discriminant analysis (OPLS-DA) with unit variance scaling, as in previous studies (33).

### Statistical Analysis

Analysis of data was performed with the Statistical Package for the Social Sciences (SPSS v22.0) (IBM, Chicago, IL, USA). Data were tested for homogeneity of variance and normality distribution by the Levene's and Shapiro–Wilk tests, respectively. When there was equality of variance and a normal distribution of data, conventional unpaired *t*-test was performed. Otherwise, the non-parametric Kruskal–Wallis test followed by the *post hoc* Mann–Whitney U (MWU) test were performed. Significant differences were established when *p* < 0.05.

Sample size estimation was calculated by the Appraising Project Office's program from the Universidad Miguel Hernández de Elche (Alicante). The minimal sample size to provide statistically significant differences among groups, using plasma IgG as variable and assuming that there is no dropout rate and type I error of 0.05 (two-sided), was three litters, as in previous studies, because of the remarkable variability among litters (34, 35).

## Results

### Growth and Fecal Characteristics

Body weight was monitored daily throughout the study (Supplementary Figure 1). Animals given 2′-FL had similar weights compared to REF animals, although at the end of the study a slightly higher body weight was found (*p* < 0.05, Supplementary Table 1). Body mass and Lee indexes were comparable between 2′-FL and REF group, whereas a higher body-to-tail-length ratio was found in the 2′-FL group on both day 8 and day 16 (Supplementary Table 1). The intake of 2′-FL did not influence the size of the organs studied (spleen, thymus, liver, and intestines), with the exception of a relatively lower colonic weight on day 16 (*p* < 0.05).

With regard to stool characteristics, supplementation with 2′-FL did not affect stool weight (REF: 4.3 ± 0.48 mg on day 8 and 7.5 ± 0.65 mg on day 16 vs. 2′-FL: 5.5 ± 0.69 mg on day 8 and 9.8 ± 0.93 mg on day 16). In addition, stool samples were scored in all cases with a score of 1 (scale 1–4) on days 8 and 16, indicating no changes in the fecal consistency due to the intervention.

### Total Immunoglobulins

Plasma concentration of Ig classes and subclasses were quantified on days 8 and 16 (Table 1). 2′-FL supplemented animals showed elevated plasma IgG concentration on day 8, primarily due to a 50% increase in IgG2b (*p* < 0.05). Accordingly, 2′-FL promoted a shift to Th1 response by increasing the Th1/Th2 immunoglobulin ratio (*p* < 0.05, IgG2b+IgG2c/IgG1+IgG2a). The Ig analysis on day 16 not only maintained the effects found on day 8, but also increased significantly the levels of IgG2a and IgG2c (*p* < 0.05). Moreover, 2′-FL supplementation raised plasma IgA levels by more than 40% compared to the REF group on day 16 (*p* < 0.05), but no effect on IgM was found.

**Table 1:** Effect of 2′-FL supplementation on the immunoglobulin levels in plasma.

	**Day 8**		**Day 16**	
**μg/mL**	**REF**	**2^**′**^-FL**	**REF**	**2^**′**^-FL**
IgM	5.65 ± 0.19	5.74 ± 0.31	19.49 ± 0.75	18.83 ± 1.15
IgG	2123.26 ± 78.15	2454.14 ± 76.43^*^	3693.28 ± 180.15	5732.33 ± 284.11^*^
IgG1	112.53 ± 3.00	105.36 ± 7.14	273.60 ± 6.67	296.84 ± 33.20
IgG2a	476.66 ± 24.12	483.95 ± 12.71	722.84 ± 17.44	884.24 ± 25.87^*^
IgG2b	558.21 ± 51.12	834.30 ± 37.10^*^	1055.80 ± 26.15	2452.3 ± 166.19^*^
IgG2c	975.86 ± 78.55	1030.52 ± 28.18	1641.03 ± 170.39	2098.97 ± 79.03^*^
Th1/Th2	2.65 ± 0.18	3.16 ± 0.05^*^	2.71 ± 0.18	3.85 ± 0.17^*^
IgA	38.88 ± 3.06	45.37 ± 1.88	63.32 ± 1.49	90.15 ± 3.37^*^

*Results are expressed as mean ± S.E.M. (n = 8/group)*.

* *p < 0.05 compared to REF (by Mann–Whitney U-test)*.

*Th1/Th2 = (IgG2b+IgG2c)/(IgG1+IgG2a)*.

*REF, Reference; 2′-FL, 2′-fucosyllactose*.

### Mesenteric Lymph Node Cell Subsets

**Figure 1:**
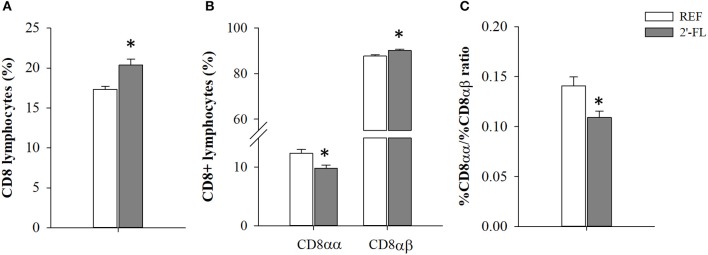
Effect of 2'-FL on CD8 surface expression in the mesenteric lymph node lymphocytes. **(A)** Percentage of lymphocytes expressing CD8. **(B)** Percentage of CD8+ lymphocytes expressing CD8αα or CD8αβ. **(C)** Ratio between the percentage of CD8αα and CD8αβ subsets. Results are expressed as mean ± S.E.M. (*n* = 4/group). *p < 0.05 compared to REF group (by Student *T*-test). REF, Reference (REF); 2′-FL, 2′-fucosyllactose.

The relative proportion of MLN cell subsets was analyzed on day 16 (Table 2). The neonatal rats administered daily with 2′-FL showed an increase in the proportion of T cells whereas that of B cells decreased (*p* < 0.05), giving rise to a deviation of the T/B ratio (*p* < 0.05, REF: 2.06 ± 0.17, 2′-FL: 4.33 ± 0.53). This increase in T cells was linked to both higher T-helper (Th) and T-cytotoxic (Tc) cell proportions (*p* < 0.05). The administration of 2′-FL increased the percentage of Th lymphocytes expressing the adhesion molecule CD62L (*p* < 0.05), but not of those Th expressing the activation marker CD25. The increase in Tc cells was both in TCRαβ+ and TCRγδ+ subsets (*p* < 0.05). Moreover, no changes were observed in the proportion of NKT and NK cell subsets and in their co-expression of CD8 in rats receiving 2′-FL as compared to control. In addition, the expression of CD8, used as overall marker of immune maturation, was also analyzed in the entire population of lymphocytes (Figure 1). In this regard, the rats receiving 2′-FL displayed a higher proportion of total CD8+ lymphocytes, which in turn were composed of a higher proportion of CD8αβ+ cells and a lower proportion of CD8αα+ cells, compared to the REF rats (*p* < 0.05). As a result, the 2′-FL group presented a lower CD8αα/CD8αβ ratio (*p* < 0.05).

**Table 2:** Effect of 2′-FL supplementation on the mesenteric lymph node immune cells proportion on day 16.

**Populations in MLN (%)**	**REF**	**2^**′**^-FL**
B cells (CD45RA+)	30.53 ± 2.54	18.13 ± 2.19^*^
T cells (Th and Tc cells)	62.01 ± 2.12	74.97 ± 2.03^*^
Th cells (CD4+ TCRαβ+ NK–)	46.21 ± 1.32	55.56 ± 1.45^*^
CD62L+	23.46 ± 3.10	29.33 ± 1.80^*^
CD25+	2.15 ± 0.53	2.64 ± 0.08
Tc cells (CD8+ TCRαβ+ NK- and TCRγδ+)	15.80 ± 0.81	19.41 ± 0.62^*^
TCRαβ+ (CD8+ TCRαβ+ NK–)	13.65 ± 0.81	16.91 ± 0.60^*^
TCRγδ+	2.15 ± 0.04	2.50 ± 0.05^*^
CD8+	1.52 ± 0.02	1.84 ± 0.06^*^
CD8–	0.62 ± 0.05	0.66 ± 0.05
NKT cells (TCRαβ+ NK+)	2.99 ± 0.46	3.08 ± 0.22
CD8+	1.72 ± 0.27	1.84 ± 0.11
CD8–	1.27 ± 0.19	1.24 ± 0.14
NK cells (TCRαβ- NK+)	3.43 ± 0.62	2.75 ± 0.16
CD8+	0.47 ± 0.11	0.50 ± 0.05
CD8–	2.96 ± 0.51	2.25 ± 0.13

*Results are expressed as mean ± S.E.M. (n = 4/group)*.

* *p < 0.05 compared to REF (by Student T-test)*.

*Th, T-helper; Tc, T-cytotoxic; NK, Natural Killer; NKT, Natural Killer T; REF, reference; 2′-FL, 2′-fucosyllactose*.

### Intestinal Gene Expression and Cytokine Production

**Figure 2:**
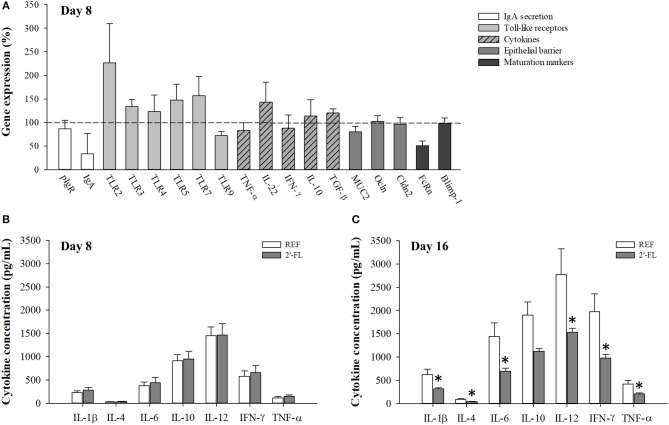
Effect of 2′-FL on the intestinal gene expression and production of cytokines. **(A)** The intestinal function was assessed on day 8 by the relative quantification of IgA secretion, Toll-like receptors, cytokines, epithelial barrier, and maturation by real-time PCR. The relative gene expression in the experimental groups was calculated with respect to REF, which corresponded to 100% of transcription (indicated with a horizontal dotted line). A collection of cytokines (IL-1β, IL-4, IL-6, IL-10, IL-12, IFN-γ, TNF-α) were quantified in the intestinal wash of animals on **(B)** day 8 and **(C)** day 16 of life. Results are expressed as mean ± S.E.M. (*n* = 8/group in **(A)**, *n* = 6/group in **(B,C)** *p < 0.05 compared to REF group (by Mann-Whitney *U*-test). pIgR, Polymeric immunoglobulin receptor; IgA, immunoglobulin A; TLR, Toll-like receptor; TNF, tumor necrosis alpha; IL, interleukin; IFN, interferon; TGF-β, tumor growth factor-beta; MUC2, mucin 2; Ocln, occludin; Cldn2, claudin 2; FcRn, neonatal constant fragment receptor; Blimp-1, B-lymphocyte-induced maturation-protein-1; REF, reference; 2′-FL, 2′-fucosyllactose.

Expression levels of genes involved in the activity of the immune system and gut barrier function were assessed on day 8 (Figure 2A). Supplementation with 2′-FL did not modify the expression of genes linked to IgA secretion (pIgR, IgA), Toll-like receptors (TLR2, 3, 4, 5, 7, and 9), cytokines (TNF-α, IL-22, IFN-γ, IL-10, TGF-β), epithelial barrier function molecules (Muc2, Ocln, and Cldn2) or the status of intestinal function (FcRn and Blimp-1). However, there was an overall trend (*p* = 0.2) toward increased expression levels of most TLR genes (with the exception of TLR4 and TLR9), with an increase in mean expression levels ranging between 20 and 125% in the 2′-FL group compared to the REF group.

Quantification of cytokines in the gut wash was performed on samples from both days 8 and 16 (Figures 2B,C). Animals who received 2′-FL displayed similar concentrations of cytokines compared to the REF animals on day 8, but not on day 16. Specifically, the 2′-FL group displayed lower levels of almost all cytokines quantified (IL-1β, IL-4, IL-6, IL-12, IFN-γ, and TNF-α), which were nearly half of those levels found in the REF group (*p* < 0.05). No changes were observed in the ratio of Th1/Th2 cytokines based on the proportion of IFN-γ/IL-4 (REF: 20.58 ± 1.08 on day 8 and 23.13 ± 8.78 on day 16; 2′-FL: 20.91 ± 0.34 on day 8; and 23.59 ± 4.36 on day 16).

### Intestinal Histomorphometry

**Figure 3:**
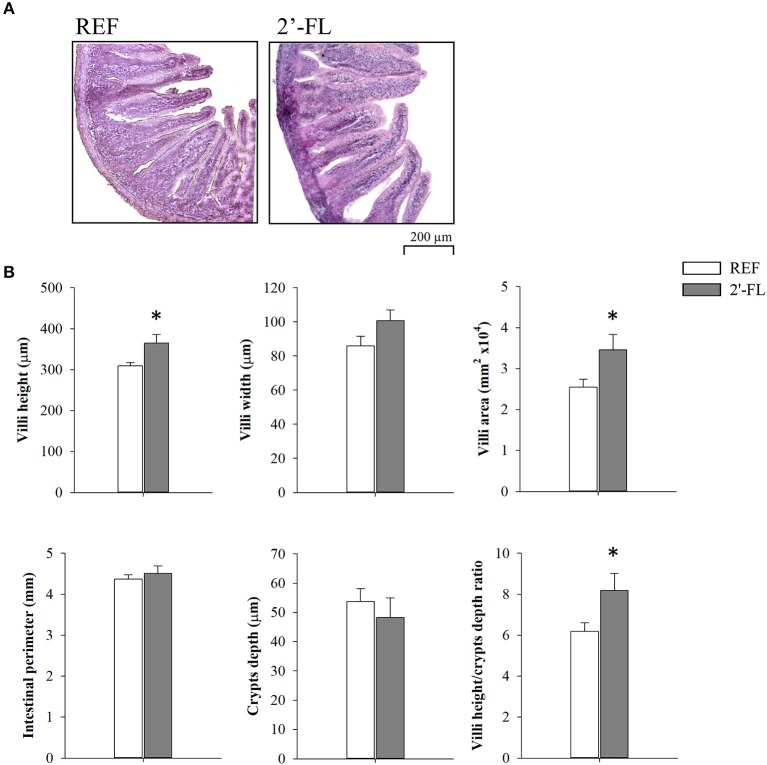
Effect of 2′-FL on intestinal histomorphometric variables on day 8. **(A)** Representative images of distal jejunum sections stained with hematoxylin and eosin, 100×. **(B)** Villi height, villi width, villi area, crypts depth, villi height/crypts depth ratio, and perimeter of the jejunum of suckling rats. Results are expressed as mean ± S.E.M. (*n* = 8/group). *p < 0.05 compared to REF group (by Student *T*-test). REF, Reference; 2′-FL, 2′-fucosyllactose.

The effects of 2′-FL on several intestinal histomorphometric parameters were assessed on day 8 (Figure 3). Animals receiving 2′-FL displayed higher villus heights and villus areas compared to the REF group (*p* < 0.05). Although no statistical differences were found in the villus widths, a tendency toward increased villus widths was observed after 2′-FL supplementation. Neither the intestinal perimeter nor crypts depths showed any remarkable changes, but villus/crypt ratios were increased (*p* < 0.05), primarily due to higher villus heights.

### Fecal Microbiota Composition

**Figure 4:**
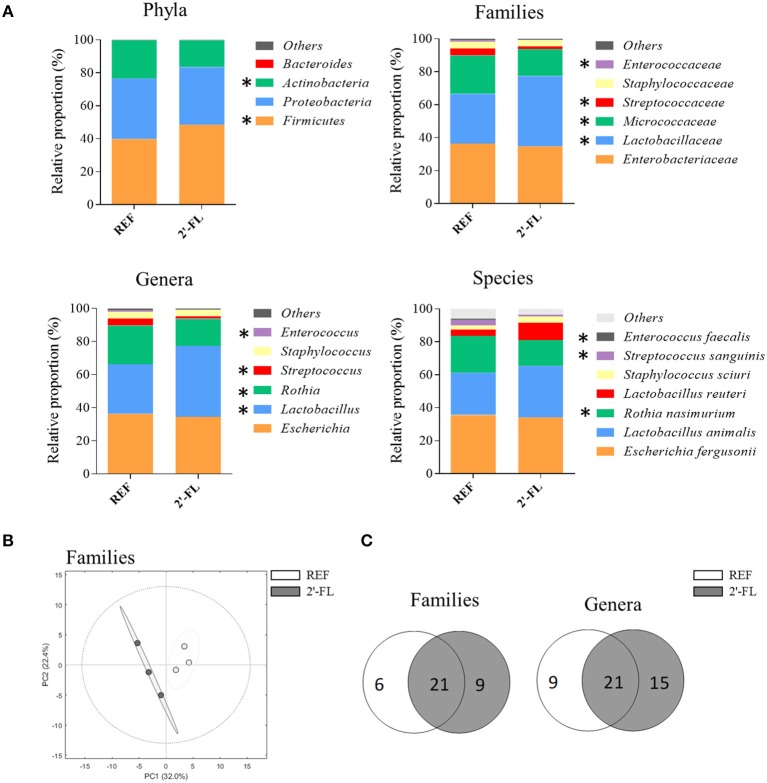
Effect of 2′-FL on the fecal microbiota composition on day 8. The sequencing of the amplicon targeting the V3–V4 region of the 16S rRNA was performed following the 16S Metagenomic Sequencing Library Illumina 15044223 B protocol. The relative proportion of the bacteria was calculated for **(A)** phylum, family, genus, and species. **(B)** PCA of the proportion of bacteria at the level of family. **(C)** Venn Diagrams showing the number of families and genera in the REF and 2′-FL groups. Results are expressed as the mean value (*n* = 3/group, corresponding to one random animal/litter). *p < 0.05 compared to REF group (by Mann–Whitney *U*-test). REF, Reference; 2′-FL, 2′-fucosyllactose.

The fecal microbiota composition was studied on day 8 (Figure 4 and Supplementary Figure 2). The diversity of microbial populations, assessed with the Shannon–Wiener and CHAO1 indexes, did not show any significant differences between the REF and the 2′-FL groups (REF: 1.68 ± 0.08 and 260 ± 50, 2′-FL: 1.63 ± 0.03, and 200 ± 17, respectively). The supplementation with 2′-FL displayed changes in bacterial proportions at the taxonomic levels of phylum, family, genera, and species (Figure 4A). At the level of phylum, the proportion of *Actinobacteria* was lower, while that of *Firmicutes* was higher in the 2′-FL group compared to the REF group (*p* < 0.05). On the one hand, the increase in *Firmicutes* was primarily due to an increase in the *Lactobacillaceae* family, in particular *Lactobacillus* genus (*p* < 0.05). In this regard, many of the *Lactobacillus* species already present in the REF group displayed a tendency to increase their levels after dietary supplementation with 2′-FL, and in addition, nine new species of *Lactobacillus* appeared (Supplementary Figure 2). On the other hand, the decrease in *Actinobacteria* was linked to a lower proportion of *Rothia*, belonging to the *Micrococcaceae* family (*p* < 0.05, Figure 4A) and to the absence of nine species, which were only found in the REF group (Supplementary Figure 2). Moreover, a decrease in less abundant families of *Firmicutes*, such as *Enterococcaceae* and *Streptococcaceae*, was observed after the supplementation with 2′-FL (*p* < 0.05). At the level of species, *Escherichia fergusonii* and *Lactobacillus animalis* were the most abundant populations in both REF and 2′-FL groups, accounting for ~80% of the total fecal microbiota.

The PCA at the level of families (Figure 4B) revealed that the animals supplemented with 2′-FL clustered apart from those of the REF group, as depicted by the lack of overlapping in the Hotelling's T2 95% confidence ellipses, indicating that the supplementation induced a different microbiota composition.

In order to gain a deeper understanding of the presence or the absence of bacterial groups after 2′-FL supplementation, Venn diagrams were represented for the number of families and genera (Figure 4C). There was a core of 21 families and genera that were present in both groups. Nevertheless, the supplementation with 2′-FL promoted the colonization with nine families and 15 species, like the previously mentioned *Lactobacillus*, and prevented those of six families and nine genera compared to the REF group. Some butyrate-producing bacteria, such as *Roseburia, Ruminococcus*, and *Blautia* appeared only after the supplementation with 2′-FL (Supplementary Figure 2).

### Cecal SCFA Production

**Figure 5:**
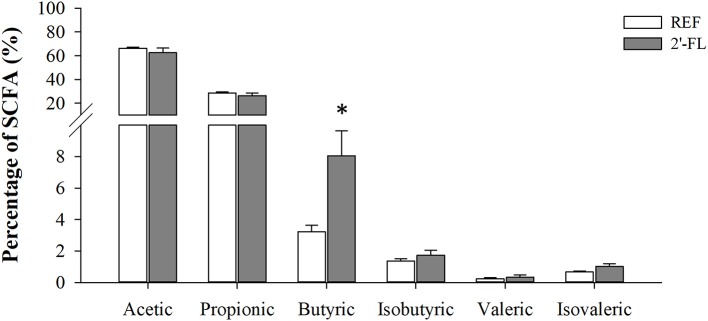
Effect of 2′-FL on the cecal SCFA production on day 16. Acetic, propionic, butyric, isobutyric, valeric, and isovaleric acid production was quantified by HS-GC-MS. Results are expressed as the mean relative percentage ± S.E.M. (*n* = 8/group). *p < 0.05 compared to REF group (by Mann-Whitney *U*-test). REF, Reference; 2′-FL; 2′-fucosyllactose.

The quantification of SCFAs in the cecal content was performed at the end of the study (day 16), enabling the changes after the 15-day supplementation to be observed (Figure 5). The 2′-FL group displayed lower total SCFA levels compared to the REF group (9.7 ± 1.89 and 17.1 ± 1.05 μM/g, respectively), mainly linked to a reduction of acetic and propionic acids (*p* < 0.05, REF: 11.35 ± 0.77 and 4.85 ± 0.30 μM/g; 2′-FL: 6.10 ± 1.4 and 2.56 ± 0.49 μM/g, for acetate and propionate, respectively). Besides that, when calculating their relative proportion (Figure 4), all SCFAs displayed similar values compared to the REF group, with the exception of butyric acid, the proportion of which was two-fold greater in the 2′-FL group than in the REF animals (*p* < 0.05).

### Metabolomic Analysis of Urine

**Figure 6:**
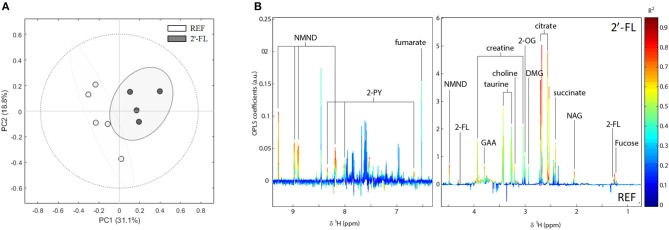
Effect of 2′-FL on the urinary metabolic profile on day 16. **(A)** NMR spectra was analyzed by natural clustering by PCA. **(B)** OPLS-DA coefficient plot showing the most significant metabolites. In this plot, positive peaks represent metabolites excreted at higher concentrations in urines of rats which received 2′-FL supplementation compared to the REF group, whereas negative peaks indicate metabolites excreted in higher concentration in the REF group (*n* = 4–5/group). REF, Reference; 2′-FL, 2′-fucosyllactose.

The metabolomic analysis of urine was performed on day 16. Initially, an unsupervised analysis was performed to obtain a general overview of the natural data grouping and to show similarities in metabolic profiles (Figure 6). A four-component PCA model from all urine samples explained 77% of the variation in the data and the scores plot of the two first principal components showed an apparent separation between REF and 2′-FL animals.

Metabolites significantly contributing to separation between REF and 2′-FL were identified from the OPLS-DA coefficients loading plot (Figure 6). Rats receiving 2′-FL excreted significantly higher amounts of tricarboxylic acid (TCA) cycle metabolites (succinate, citrate, 2-oxoglutarate [2-OG], and fumarate), metabolites from the nicotinate and nicotinamide pathway (1-methylnicotinamide [NMND] and N-methyl-2-pyridone-5-carboxamide [2-PY]), gut microbial metabolites of choline (choline and dimethylglycine [DMG]), creatine-related metabolites (creatine, guanidinoacetate [GAA]), taurine, N-acetyl glycoproteins (NAG), and those related to the oligosaccharide (fucose and 2′-fucosyllactose). Metabolites identified as contributing to separation between treatments through the OPLS-DA model were integrated and spectral peak integrals were further analyzed for statistical significance using univariate methods (Supplementary Table 2).

## Discussion

There is evidence supporting the beneficial effects of HMOs in early life, for example by modulating the immune system, changing the microbial composition, or protecting against infections (4, 36, 37). The present study gained insights into the effects of 2′FL supplementation, showing improved maturation of the immune system, gut trophic effects and as well as supporting the development of a balanced gut microbiome. The nutritional intervention with 2′-FL did not show biologically relevant changes in the growth of the animals compared to the REF group, as similarly evidenced in other studies (18, 19, 26, 38). In addition, the relative weights of the organs analyzed and the stool characteristics were not affected after 15 days of supplementation, confirming that their administration was safe and well-tolerated, which is in line with studies performed by other authors (26, 39). The histomorphometric analysis of the small intestine revealed that administration of 2′-FL for 7 days had an intestinal trophic effect as observed by higher villus heights and areas. Conflicting results have been reported by others showing higher villus heights after administering 2′-FL to newborn pigs infected with *E. coli* (40) or after extensive intestinal resection in mice (41). Other studies did not find any alterations of the gut histomorphometry after 2′-FL supplementation in RV-infected piglets (42) or in preterm pigs (43). Overall, the present study showed positive effects on intestinal growth after 2′-FL supplementation which might impact the absorptive capacity of the animals.

Human milk oligosaccharides (HMOs) are known to influence the mucosal and systemic immunity, either directly by interacting with the immune cells, or indirectly, for example through the microbiota (36). We assessed the effects on the immune system with regard to both the humoral and cellular arms after the supplementation with 2′-FL. The animals supplemented with the oligosaccharide already displayed higher levels of circulating IgGs on day 8. This effect was stronger on day 16, when in addition to the higher IgGs, an increase in IgA was also detected. A recent study has also evidenced an increased IgG response after the vaccination of mice supplemented with 2′-FL with influenza virus (44). When considering the IgG subclasses, it was observed that 2′-FL supplementation promoted an increase in the Th1-type immunoglobulins compared to Th2-type, increasing the Th1/Th2 ratio. Accordingly, at birth, the immune system is biased toward a Th2 phenotype, which is linked to promoting humoral responses, as opposed to that of the immature cellular immunity (45). As the immune system matures, the response switches toward a Th1 phenotype (46). Therefore, the increase in Th1 immunoglobulins would suggest that 2′-FL promotes the maturation of the immune system in early life.

Because the major effects of 2′-FL are supposed to be exerted in the intestine or its surroundings, we evaluated the immune cell subsets in the MLNs and the intestinal production of cytokines. Herein, we have shown that the HMO 2′-FL promoted higher T cell and lower B cell proportions in MLNs on day 16 of life. It can be suggested that B cells may be migrating from the MLN to the systemic compartment or becoming plasma cells, because despite observing a relative decrease in B cell proportion in MLN, this was not linked to a decrease in the production of Ig, as their circulating levels were elevated. In fact, the values displayed by the animals receiving the oligosaccharide more closely resembled those also found in healthy Lewis suckling rats of 19 days of life (47), suggesting that 2′-FL may accelerate intestinal immune cell maturation. The rise in T cell proportion was due to both Th and Tc cells increases, further supporting the concept that 2′-FL drives the age-related switch to Th1 phenotype, since Tc cells are generated with the aid of this type of response. A particular subset of Tc cells, γδ T cells, pre-dominate during fetal and early life, and are important for mounting rapid innate responses against exogenous pathogens in the gut (48, 49). The CD8+ γδ T cell subset proportion was increased after the supplementation with 2′-FL, again supporting the idea that the oligosaccharide influences immune maturation. This was in agreement with Grases-Pintó et al., who found an increase of this subset in MLNs of rats aged 14–21 days (50). On the contrary, Comstock et al. did not find any differences in Th or Tc subsets in pigs fed an HMO mixture containing 2′-FL, but found increased proportions of memory effector T cells in MLNs of 15-day-old piglets (38). Moreover, recent data supports that 2′-FL improves innate and adaptive cellular immunity, for example by expanding vaccine-specific splenic CD4+ and CD8+ T cells and inducing optimal Th1 responses (44). Finally, the increase in the total CD8 lymphocytes and the decreased CD8αα/CD8αβ ratio, biomarkers of intestinal immune maturation described in previous studies (50), also evidence that 2′-FL promoted an accelerated intestinal immune maturation.

We observed a general decrease of intestinal cytokines on day 16, but not on day 8. This evidenced that immunomodulation at the cellular level was either depending on the 2′FL dose, as the total amount received on day 16 is higher than on day 8, or that the effects are time-dependent. Several of the cytokines that were reduced after 2′-FL supplementation are highly inflammatory (IL-1β, IL-6, IFN-γ), and this therefore supports the anti-inflammatory potential of the oligosaccharide. In fact, a randomized controlled trial found that circulating plasma concentrations of these cytokines are higher in standard formula-fed infants, and that infants fed a formula containing 2′-FL have lower concentrations of inflammatory cytokines at comparable levels to those found in breastfed infants (6).

The microbiota of the newborn is characterized by low diversity and rapid transformation. Facultative aerobic species colonize the intestine initially, and as the oxygen is consumed, they are replaced by anaerobic species (51–53). Herein, we have evidenced that the fecal microbiota of 8-day-old rats was dominated by the phyla *Firmicutes, Proteobacteria*, and *Actinobacteria*, with *Escherichia, Lactobacillus*, and *Rothia* being the genera accounting for more than 90% of the microbiota. The PCA analysis revealed that those individuals who received 2′-FL displayed a different microbiota compared to those that did not, since both experimental groups clearly clustered apart.

We aimed to assess the pre-biotic effect of 2′-FL and found an increase in *Lactobacillus*, but not in *Bifidobacterium* proportions. Because HMOs are not extensively degraded by β-glucosidases of lactobacilli (54), their increase does not seem to involve direct catabolism. Studies using purified HMOs demonstrate that most lactobacillus do not utilize 2′-FL as a carbon source (55), although some strains such as *L. acidophilus, L. plantarum*, and *L. mesenteroides* might be able to hydrolyze it weakly (56). Therefore, it remains unclear the exact mechanism of the pre-biotic effect of 2′-FL in the *Lactobacillus* population; some other hypotheses, such as the changes observed in the host's immune system, microbial cross-feeding interactions or SCFA production, may play a significant role. It is well-documented that HMOs can be degraded by many strains of *Bifidobacterium*, which are known to take part in the healthy microbiota of the infant (57–59). However, the microbiota of the 8-day-old rat showed a small proportion of these bacteria, most likely because *Bifidobacterium* is a strictly anaerobe and it probably appears later in life. Moreover, numbers of bifidobacteria seemed to start increasing after approximately 14 days of supplementation (60), which could explain why we did not find any differences earlier. Nevertheless, 2′-FL showed a pre-biotic effect by increasing the proportion of lactobacilli.

We also found a decrease of other facultative anaerobes from the genera *Streptococcus* and *Enterococcus*. This fact, together with a higher lactic acid bacteria, could suggest that the microbiota is changing more rapidly after 2′-FL supplementation, since after the first week of life, some genera such as *Staphylococcus* and *Enterococcus* are known to decrease (60).

The present study also aimed to assess the metabolic changes after 2′-FL by means of assessing the intestinal SCFA production and the urinary metabolomic profile of the rats. The bacterial end-product SCFAs are produced after the metabolism of HMOs in the gut (13, 61). Herein, we found that there was a reduction in the concentration of total SCFAs in the 2′-FL group, which may be due to lower production or to a higher absorption. However, the relative amount of butyric acid in cecal content was remarkably increased. Bifidobacteria are known to be one of the main producers of lactate and acetate, the main SCFAs (13, 62). The low counts of these types of bacteria may explain why we did not find an increase in the total SCFAs. Besides, butyrate is well-known for its health-promoting properties in the gut and has been shown to be elevated after the supplementation with HMOs (37). This SCFA may be involved in immune homeostasis, for example by reducing pro-inflammatory pathways, such as STAT, AP-1, and NF-κB (63, 64). Therefore, the increased butyrate may be associated with the anti-inflammatory properties of 2′-FL found in the present study as evidenced by reduction of intestinal cytokine levels. In addition, the increase in butyrate after 2′-FL supplementation could be related to the higher excretion of tricarboxylic acid (TCA) cycle intermediates observed in the urines of this group. Furthermore, butyrate has been shown to be a histone deacetylase (HDAC) inhibitor and thus potentially increases the acetylation of a wide number of metabolic proteins, and nearly every enzyme involved in the TCA cycle is acetylated (65). It can also act as an energy source, undergoing β-oxidation to acetyl-Coa in the colonocytes, which enters the TCA cycle. The metabolomic analysis also revealed that in both groups 2′-FL was partially absorbed and excreted unaltered in urine, indicating that Lewis rats are secretory research animal models, as has been reported previously (2, 66). Moreover, a higher content of fucose was detected in urine samples of the 2′-FL group, suggesting that the fucosidases of the microbiota efficiently liberated the monosaccharide and this was later absorbed in the colon. Indeed, several enteric bacteria have been shown to liberate fucose after HMO degradation. However, although most of them are able to metabolize the galactose and glucose forming part of the lactose, they might not be able to process fucose, which is liberated and subsequently absorbed by the gut (67, 68). Besides microbial metabolites, other endogenous molecules were also present in higher proportions in the urine, such as the metabolites of the TCA cycle, indicating that 2′-FL also has an influence on the host metabolism. Alternatively, considering the possibility that SCFAs enter the TCA cycle of the colonic enterocytes and that these metabolites produced in the gut end up in the urine, the changes in the SCFA found by 2′-FL can also influence these TCA cycle metabolites.

The mixture of oligosaccharides found in human milk has been shown to influence gut immunity and microbiome. In addition, it has to be taken into account that the administration of a single bolus dose of 2′FL performed in this study does not represent the typical exposure produced during breastfeeding, and therefore, the obtained results should not be directly extrapolated. Although a single HMO cannot mimic the structural and functional complexity of the mixture, the effects of individual oligosaccharides are relevant to be further elucidated for future application to improve health in early life. In the present study, the HMO 2′-FL was identified as having multiple health benefits in early life. The relatively immature immune system of the newborn was shown to be more developed and probably more prepared to fight infections, as depicted by a Th1-biased phenotype in both cellular and humoral compartments. Moreover, 2′-FL seems to promote immunoregulatory effects, as it decreased the levels of inflammatory cytokines in the gut. The higher *Lactobacillus* proportion in the fecal microbiota and the higher proportion of the SCFA butyrate supports the pre-biotic role of this oligosaccharide. Finally, 2′-FL not only induced local effects, such as a trophic effect in the small intestine, but it was also able to be metabolized by the microbiota, to arrive at the systemic compartment and change the urinary metabolic profile. Future experiments should evaluate the relationship between the intestinal and systemic effects found here, as well as gain a deeper insight into the host–microbiota crosstalk, in order to decipher the mechanisms of action of 2′-FL, other single HMO, but also the whole complex HMO mixture, in early life.

## Data Availability

The datasets generated during and/or analyzed during the current study are available from the corresponding author on reasonable request.

## Ethics Statement

This study was carried out in accordance with the recommendations of EU-Directive 2010/63/EU for the protection of animals used for scientific purposes. The protocol was approved by the Ethical Committee for Animal Experimentation of the University of Barcelona and the Catalonia Government (CEEA-UB Ref. 74/05 and DAAM 3046, respectively).

## Author Contributions

IA-B, MM-C, JM-P, KK, BvL, ST, BS, JG, ÀF, MC, MR-L, and FP-C were involved in the design and/or execution of the experiments. IA-B and FP-C analyzed and interpreted the results and drafted the paper. All authors read and approved the final version of the manuscript for publication and contributed to the critical revision of the manuscript.

### Conflict of Interest Statement

The authors declare that they have a financial relationship with the organization that sponsored the research. KK, BvL, ST, BS, and JG are employees of Danone Nutricia Research. JG is head of the Division of Pharmacology, Utrecht Institute for Pharmaceutical Sciences, Faculty of Science at the Utrecht University, and partly employed by Danone Nutricia Research. BvL as indicated by the affiliations, is leading a strategic alliance between University Medical Centre Utrecht/Wilhelmina Children's Hospital and Danone Nutricia Research. The remaining authors declare that the research was conducted in the absence of any commercial or financial relationships that could be construed as a potential conflict of interest.
