# Supplementary material for: Immunomodulatory and Prebiotic Effects of 2′-Fucosyllactose in Suckling Rats
Source: Front Immunol. 2019 Jul 31;10:1773. doi: 10.3389/fimmu.2019.01773 (PMC6685134; doi:10.3389/fimmu.2019.01773)
Supplement: Supplementary file 1 [file Data_Sheet_1.PDF]

SUPPLEMENTARY MATERIAL

Supplementary Table 1. Effect of 2'-FL supplementation on growth variables

		Day 8		Day 16	
		**REF**	**2'-FL**	**REF**	**2'-FL**
**Body size**	**Body weight (g)**	13.52 ± 0.25	13.55 ± 0.27	31.40 ± 0.89	33.93 ± 0.50*
	**Body/tail length ratio**	2.14 ± 0.07	2.28 ± 0.04*	1.77 ± 0.02	1.86 ± 0.02*
	**BMI (g/cm^2)**	0.30 ± 0.01	0.29 ± 0.01	0.35 ± 0.01	0.37 ± 0.01
	**Lee Index (g$^{0.33}$/cm)**	354.76 ± 3.42	346.63 ± 4.23	334.44 ± 2.51	335.94 ± 1.88
**Organ size**	**Spleen (w/w, %)**	0.57 ± 0.03	0.63 ± 0.02	0.47 ± 0.02	0.52 ± 0.03
	**Thymus (w/w, %)**	0.34 ± 0.01	0.29 ± 0.02	0.41 ± 0.02	0.42 ± 0.03
	**Liver (w/w, %)**	2.97 ± 0.12	2.99 ± 0.14	3.65 ± 0.09	3.60 ± 0.11
	**Small intestine (w/w, %)**	3.10 ± 0.06	3.22 ± 0.11	2.88 ± 0.06	3.08 ± 0.09
	**Large intestine (w/w, %)**	0.60 ± 0.01	0.63 ± 0.05	1.07 ± 0.03	0.99 ± 0.01*
	**Small intestine length (cm)**	25.33 ± 0.73	26.70 ± 0.56	32.64 ± 1.54	32.45 ± 1.66
	**Large intestine length (cm)**	4.75 ± 0.08	4.52 ± 0.15	6.68 ± 0.17	6.49 ± 0.25

Results are expressed as mean ± S.E.M. (n=8-12/group).
*p<0.05 compared to REF (by Mann-Whitney U test).
Body mass index (BMI), weight/weight (w/w), reference (REF), 2'-fucosyllactose (2'-FL).

Supplementary Table 2. Metabolomic analysis of urine. H-NMR assignments, correlation values, and normalized peak integrals of metabolites identified by OPLS-DA model.

Metabolite	δ (ppm) and multiplicity[1]	R^2	REF	2’-FL	P[3]
**Fucose**	1.21(d), **1.25(d)**, 3.45(dd), 3.64(dd), 3.79(m), 3.88(dd), 4.20(q), 4.56(d), 5.21(d)	0.808	27.2 ± 1.7	31.0 ± 2.4	0.027
**2’-Fucosyllactose**	1.26(d), **4.26(m)**, 4.52(d), 4.63(d), 5.22(d)	0.965	10.3 ± 0.9	13.5 ± 1.0	<0.001
**NAG**	**2.04(s)**	0.856	53.5 ± 2.4	66.1 ± 8.7	0.059
**Succinate**	**2.41(s)**	0.861	83.5 ± 11.4	107.4 ± 5.9	0.007
**Citrate**	**2.54(d)**, 2.66(d)	0.902	826.1 ± 77.6	1050.8 ± 50.5	0.002
**2-OG**	2.45(t), **3.01(t)**	0.687	218.4 ± 24.6	250.7 ± 24.2	0.089
**Fumarate**	**6.53(s)**	0.720	5.1 ± 1.5	7.4 ± 2.2	0.111
**DMG**	**2.93(s)**, 3.72(s)	0.631	39.9 ± 9.6	51.2 ± 7.0	0.081
**Choline**	**3.21(s)**, 3.52(m), 4.07(m)	0.836	31.9 ± 4.1	48.5 ± 13.1	0.030
**Taurine**	3.27(t), **3.43(t)**	0.778	19.5 ± 7.8	53.8 ± 29.8	0.041
**Creatine**	**3.03(s)**, 3.94(s)	0.796	56.1 ± 11.0	89.9 ± 24.8	0.028
**GAA**	**3.80(s)**	0.785	45.8 ± 9.3	59.0 ± 3.9	0.034
**NMND**	**4.48(s)**, 8.19(t), 8.9(d), 8.97(d), 9.28(s)	0.871	39.3 ± 4.8	55.2 ± 9.9	0.016
**2-PY**	3.65(s), 6.67(d), 7.97(dd), **8.34(d)**	0.900	1.35 ± 0.20	1.91 ± 0.30	0.012

Results are expressed as normalized peak integrals of metabolites in mean arbitrary units ± S.D. (n=4-5).
[1]Signal intensities calculated from the area under curve for distinct NMR resonances (bolded) arising from metabolites of interest.
[2]Regression coefficient obtained from the OPLS model comparing REF *vs*. 2’-FL.
[3]Independent samples t-test.
N-acetyl glycoproteins (NAG), 2-oxoglutarate (2-OG), dimethylglycine (DMG), guanidoacetate (GAA), 1-methylnicotinamida (NMND), N-methyl-2pyridone-5-carboxamide (2-PY), singlet (s), doublet (d), triplet (t), quartet (q), multiplet (m), doublet of doublets (dd), reference (REF); 2’-fucosyllactose (2’-FL).

It can be observed that rats supplemented with 2’-FL showed higher urinary excretions of metabolites involved in the one-carbon metabolism, in particular, choline, DMG, NMND, and 2-PY. Choline is a major source of methyl groups used to generate the body’s main methylating agent, S-adenosylmethionine (SAM). Hence, betaine, a product of choline oxidation, is converted to DMG, as it methylates homocysteine to methionine; this is subsequently used to generate SAM, which is involved in multiple methylation reactions including epigenetic processes. Choline deficiencies have been suggested to produce epigenetic changes in genes linked to atherosclerosis and brain development. Although SAM concentrations were not measured, the increase in the urinary excretions of NMND, 2-PY and creatine suggests a regulation of the one-carbon metabolism pathway. On the one hand, NMND is formed from the methylation of nicotinamide by the SAM-dependent enzyme, nicotinamide N-methyltransferase (NNMT), which is further metabolized to 2-PY. In addition, NMND was found to have anti-inflammatory properties. On the other hand, the end-product of DMG catabolism is glycine, which can react with arginine in the kidney to synthesize GAA, which can be methylated in the liver by the SAM-dependent enzyme guanidinoacetate N-methyltransferase (GAMT) to yield creatine. In addition, the higher creatine excretion could reflect an

This is a provisional file, not the final typeset article

increasing muscle mass, which could explain the higher body weight observed in the rats supplemented with 2'-FL. Our results are consistent with the findings of previous studies showing an alteration of the transmethylation metabolic pathways in liver and pancreas with a prebiotic intervention. Hence, Martin et al. found higher choline and DMG levels in the liver, suggesting stimulated transmethylation in the methionine cycle. Finally, taurine, which is considered an essential aminoacid for brain development in newborns, was excreted in higher amounts in the urine of 2'-FL-treated suckling rats. It is synthesized from cysteine and methionine, which brings additional evidence of a stimulation of the one-carbon metabolism pathway.

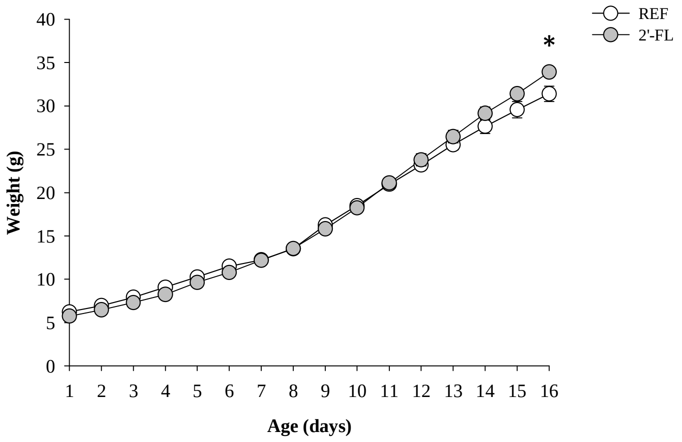

Supplementary Figure 1. Body weight of neonatal rats during 1 to 16 days of life. Results are expressed as the mean relative percentage ± S.E.M. (n=8-24/group). *p<0.05 compared to REF (by Mann-Whitney U test). Reference (REF), 2’-fucosyllactose (2’-FL).

This is a provisional file, not the final typeset article

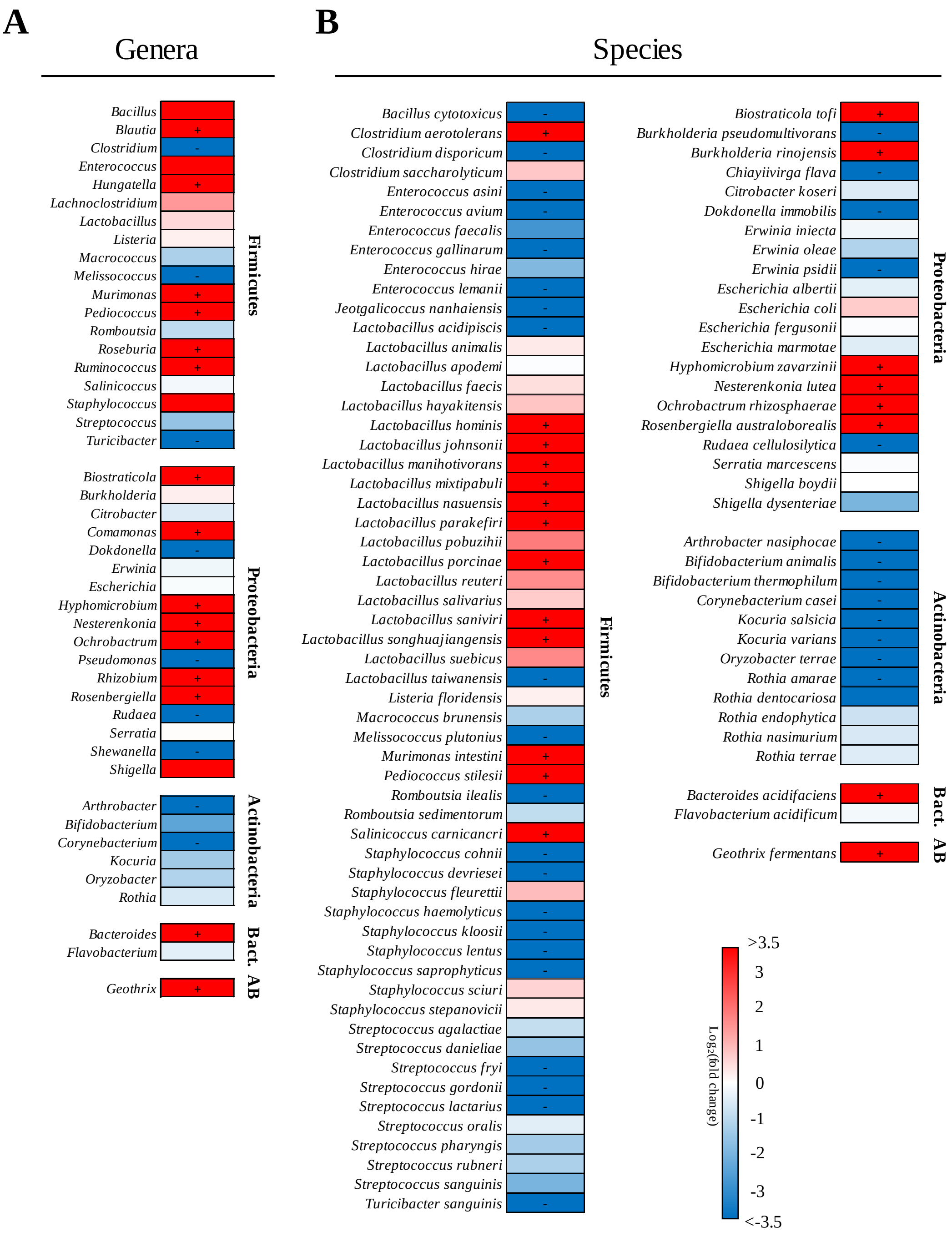

Supplementary Figure 2. Summary of the variation of genera and species in fecal microbiota on day 8. A heat map of the mean relative abundances of (A) genera and (B) species is represented. The rows represent the taxonomic classification and the columns the $\log_2$ of the fold change in the 2'-FL group with respect to REF, following the color scale of the legend. The taxonomic ranks which were present in a group but not in the REF group were assigned the maximum variation (>3.5, marked with a "+"), whereas those which were present in REF group and not in the other groups were assigned the minimum variation (<3.5, marked with a "-"). Results derived from n=3/group, corresponding with 1 random animal/litter. *Bacteroidetes* (Bact.), *Acidobacteria* (AB).
